# Uterocutaneous fistula following cesarean section: a case report

**DOI:** 10.3389/frph.2026.1847520

**Published:** 2026-06-04

**Authors:** Shahd Khatib, Nardeen Hammad, Shahd Al Aloul, Waleed Malhes, Ibaa Barghouthi

**Affiliations:** 1 Faculty of Medicine, Al-Quds University, Jerusalem, Palestine; 2 Palestine Medical Complex, Ramallah, Palestine

**Keywords:** caesarean complications, fistula, gyne and obstetrics, gyneacological surgery, uterocutaneous fistula

## Abstract

This case report details the clinical journey of a 37-year-old woman, gravida 6 para 6 + 1, who presented with persistent purulent discharge from her cesarean section scar following an elective cesarean delivery. Her obstetric history included two uncomplicated vaginal deliveries and four cesarean sections, with significant complications in the previous pregnancies, including gestational diabetes and placenta previa. Notably, ultrasound findings during her most recent pregnancy raised concerns for placenta accreta spectrum. Despite a vertical midline incision for cesarean delivery and intraoperative findings confirming placenta accreta, the patient developed a chronic purulent discharge approximately two months post-surgery, resistant to multiple courses of antibiotics. Evaluation revealed a hypoechoic area and CT imaging suggested postoperative scarring. Pelvic MRI confirmed the diagnosis of a uterocutaneous fistula (UCF). Management initially involved antibiotic therapy; however, surgical intervention became necessary due to persistent symptoms. During surgery, extensive adhesions were identified, along with the fistulous tract. A total hysterectomy was performed without immediate complications, and the patient was discharged in stable condition within a week. This case underscores the complexities of managing postoperative complications in patients with significant obstetric histories and highlights the importance of multidisciplinary approaches in challenging surgical scenarios.

## Background

Obstetric fistulas, a particularly serious and frequently debilitating condition, affect women during childbirth especially the uterine fistulas commonly occur between the uterus and adjacent organs such as the urinary bladder or the colon, the rarest form is the uterocutaneous fistula which represent an abnormal tract formation between the uterus and skin surface. This condition primarily results from obstructed labor, a complication marked by prolonged and unsuccessful attempts to deliver a baby, potentially leading to considerable maternal morbidity and mortality (1). The clinical presentation of uterocutaneous fistula is variable, the main characteristic is cyclical bleeding through the cutaneous opening synchronized with menstruation, persistent discharge from the abdominal scar,poorly wound healing, purulent discharge, and signs of local infection or inflammation also common (2). Multiple,complicated previous cesarean section,myomectomy,endometritis, surgical site infection,poor wound healing,retained surgical material, complicated labor,play a major role in development of the fistula.

**Figure 1:**
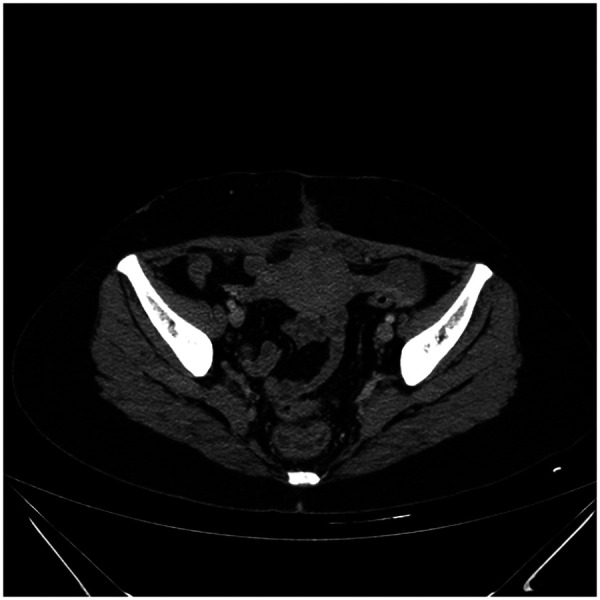
_CT._

**Figure 2:**
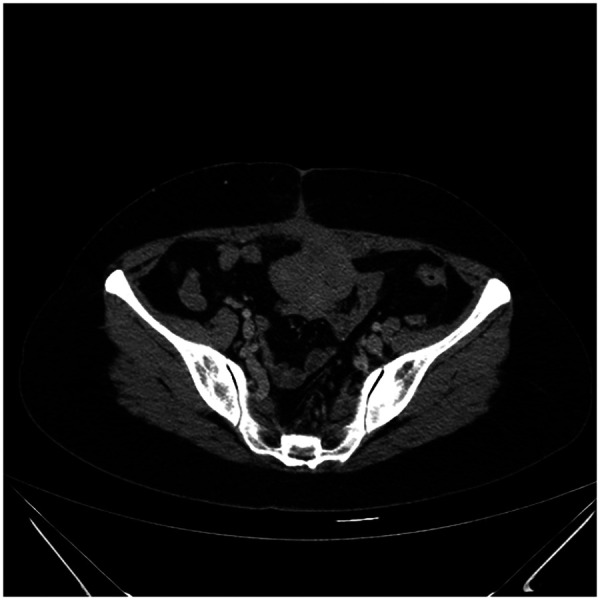
_MRI._

Recent literature continues to describe uterocutaneous fistula as an extremely rare condition,with approximately 100–150 cases reported worldwide to date, it accounts for less than 1% of all reported genital fistula cases (1). Women afflicted by obstetric fistulas frequently experience a spectrum of adverse effects, encompassing harm to reproductive structures, persistent infections, and neurological deficits, which in turn precipitate profound emotional suffering and social exclusion. These women often report constant leaking of urine and feces, along with genital discomfort and unpleasant smells, even though they try to keep themselves clean (3).

Diagnosis of uterocutaneous fistula relies on a high index of clinical suspicion, imaging plays a crucial role in confirmation, MRI provides the most precise assessment of the fistulous tract and anatomical extent. Adjunctive modalities such as hysterosalpingography and fistulography can further demonstrate the communication between the uterine cavity and the cutaneous opening,the management can be broadly classified into medical and surgical, medical treatment is mainly include antibiotics in cases of associated infections,along with hormonal suppression using GnRH(GnRHa) to induce temporary uterine quiescence and reduce menstrual flow,the definitive treat is surgical,involving complete excision of the fistulous tract (4).

## Case presentation

A 37-year-old woman, Gravida 7 para 6 abortus 1 (G7 P6 A1), presented with a persistent purulent discharge from her cesarean section scar. Written informed consent was obtained from the patient for publication of this case report and accompanying image. Her obstetric history included two prior uncomplicated term vaginal deliveries, followed by four cesarean sections. Notably, the third pregnancy was complicated by gestational diabetes mellitus (GDM) and cesarean section was performed due to breech presentation and complicated by gestational diabetes mellitus (GDM). Additionally, she had a first-trimester abortion managed via dilation and curettage and a history of schwannoma excision, as well as receiving blood transfusions during previous pregnancies.

During the most recent pregnancy, early ultrasound confirmed a viable intrauterine fetus with normal growth. However, at 34 weeks' gestation, antenatal transvaginal ultrasound raised suspicion of placenta accreta spectrum due to an anterior placenta previa overlying the previous cesarean scar with concerning sonographic features. The patient was subsequently referred to the fetomaternal unit for further evaluation. Doppler ultrasound assessment revealed no evidence of abnormal placental invasion or bridging vascular channels, and thus placenta accreta spectrum remained a suspected but unconfirmed diagnosis.

During cesarean section, the placenta was delivered smoothly and completely with easy separation from the uterine wall, without evidence of abnormal adherence, thereby effectively excluding placenta accreta intraoperatively. However, bleeding was encountered from the placental bed, which was controlled successfully with uterine suturing and standard hemostatic measures. Intraoperative findings were therefore not consistent with the placenta accreta spectrum.

Approximately two months after surgery, She developed cyclic blood-stained discharge from the surgical site, The discharge continued for nearly three months despite multiple courses of targeted outpatient antibiotic therapy. Notably, the discharge became bloody during menstruation and reverted to a serosanguinous or purulent character afterward. She denied fever, abdominal pain, or other systemic symptoms.

Initial wound cultures demonstrated methicillin-resistant *Staphylococcus aureus* (MRSA) sensitive to vancomycin and teicoplanin. Despite multiple rounds of targeted outpatient antibiotics, the discharge persisted. Upon admission for further evaluation, the patient was afebrile and hemodynamically stable. Abdominal examination revealed a soft, non-tender abdomen with a 1-cm soft area just below the umbilicus, from which a serosanguinous discharge was noted. Laboratory investigations showed mild anemia and elevated inflammatory markers.

Ultrasound revealed a hypoechoic area between the uterus and anterior abdominal wall. Contrast-enhanced computed tomography suggested postoperative scarring with a possible hypodense lesion at the uterine fundus communicating with the abdominal wall Figure 1. Given the suboptimal sensitivity of CT for the diagnosis, pelvic magnetic resonance imaging (MRI) was performed, confirming the diagnosis of a uterocutaneous fistula (UCF) by revealing a linear T2-hyperintense tract extending from the uterine fundus to the anterior abdominal wall Figure 2.

Management initially included intravenous vancomycin, later switched to cefazolin based on culture sensitivities. Prophylactic enoxaparin was administered; however, purulent discharge persisted necessitated surgical intervention. The planned procedure was a fistulectomy for definitive management of the uterocutaneous fistula. The patient was extensively counseled preoperatively regarding the procedure and its potential complications, including intraoperative hemorrhage, injury to adjacent structures, failure of complete fistula tract excision, and the possible need for conversion to hysterectomy in the event of uncontrolled bleeding or extensive disease. Given that she had completed her family, informed consent was obtained with full understanding of these risks.

Intraoperatively, severe adhesions were identified between the uterus and the anterior abdominal wall. A trial of fistulectomy was initially attempted; however, it was complicated by opening of the previous cesarean scar accompanied by massive bleeding. Consequently, total hysterectomy was performed to control the hemorrhage and definitively manage the condition. Cystoscopy and hysteroscopy revealed no additional abnormalities. The patient exhibited no immediate postoperative complications and was discharged in stable condition within 7 days. At the 3-month postoperative follow-up, she remained asymptomatic with complete healing of the surgical wound and no evidence of fistula recurrence.

## Discussion

There are only a few case reports and small case series in the literature that describe uterocutaneous fistula, a rare complication that can occur after caesarean delivery. The true incidence of this condition is unknown due to its rarity (5). This abnormal connection between the uterus and the abdominal wall presents substantial diagnostic and therapeutic challenges for obstetricians and gynecologists. Due to its rarity, the condition is often overlooked, leading to delays in diagnosis and extended patient suffering, as healthcare providers may not recognize it despite its distinctive clinical signs. The risk factors for UCF are multifactorial, including multiple caesarean sections, postoperative MRSA infections, and surgical complexities associated with placenta accreta spectrum, all of which may have contributed (5, 6). In this case, the patient had previously undergone four cesarean sections, increasing her risk. Each cesarean creates potential weak points for fistula formation due to disruption of the uterine wall and complications during healing. The repeated instances of repeated caesarean scars elevate the likelihood of incomplete healing and excess scar tissue, which can eventually lead to the development of a fistula. Furthermore, the presence of placenta accreta spectrum, which was confirmed during her previous cesarean delivery, is not typically a direct cause of UCF. Instead, it likely played an indirect role by making the cesarean delivery more complex, thus increasing the potential for tissue trauma, bleeding, subsequent adhesions and fistula formation (7).The situation was made more complicated by the presence of placenta previa major anterior, which sits over a previous cesarean scar, increasing the risk of placental invasion and adherence, necessitating more extensive surgical procedures (7, 8).

The development of a uterocutaneous fistula involves a multifaceted interaction of surgical trauma, infection, inflammation, and impaired wound healing. The initial injury occurs during the cesarean section when the hysterotomy incision is made. Cases involving placenta accreta may suffer additional tissue damage during the removal of the placenta and uterine repair. Postoperative infections, such as those caused by MRSA and other gram-positive organisms identified in this patient, contribute to localized tissue necrosis and the formation of abscesses. These factors can create a pathway from the uterus to the abdominal wall (5). Chronic inflammation and granulation tissue formation can sustain the fistulous tract, preventing it from closing on its own. The defining clinical characteristic of a uterocutaneous fistula is the cyclical bleeding or blood-tinged discharge emanating from an abdominal scar, which frequently aligns with the menstrual cycle. Although a persistent purulent discharge might be observed in cases of secondary infection, as was noted in this patient, it is the cyclical pattern of the drainage that serves as the classic indicator of this condition (5, 9). The lack of systemic infection symptoms can often contribute to a delayed diagnosis. A high degree of clinical suspicion is warranted in patients who experience persistent discharge from a cesarean scar, particularly when symptoms do not respond to repeated antibiotic treatments or wound care (5, 10). Timely diagnosis is crucial, given that its infrequent occurrence and non-specific presentation often lead to delays (5, 10).

Diagnosing a suspected uterocutaneous fistula requires a combination of imaging techniques. Ultrasound is commonly the initial imaging modality, potentially revealing a hypoechoic tract or a fluid collection extending from the uterus to the abdominal wall (9, 10). While fistulography and hysterosalpingography can delineate the fistulous communication by demonstrating contrast passage, their use has diminished due to advancements in cross-sectional imaging (4, 5). Contrast-enhanced CT scans can aid in identifying inflammatory changes, abscess formation, and the anatomical course of the fistula (5, 10). However, pelvic magnetic resonance imaging (MRI) remains the most definitive diagnostic method due to its superior soft-tissue resolution, clearly visualizing the fistulous tract and surrounding pelvic structures (4, 5, 10). MRI offers enhanced detail, allowing for clear visualization of the fistulous tract as a bright line on T2-weighted images, extending from the uterine lining to the abdominal wall. In confirmed cases, pelvic MRI can definitively show a linear T2-hyperintense tract spanning from the endometrium at the uterine fundus to the anterior abdominal wall. Hysteroscopy can also be valuable for identifying the internal uterine opening, supporting preoperative assessment and surgical planning (4, 8). Other diagnostic methods, such as hysteroscopy or fistulography, can support the diagnosis but are used less frequently today because of MRI's superior accuracy (5).

The manual extraction of the placenta, necessitated by the placenta accreta spectrum, likely resulted in significant uterine wall trauma and focal devitalization of the myometrium (7). This surgical trauma, occurring in the setting of multiple previous cesarean sections, created a weakened uterine segment that was highly susceptible to impaired healing (11). The subsequent development of a postoperative MRSA infection further exacerbated tissue necrosis, providing a pathway for the formation of the fistulous tract between the uterus and the abdominal wall (5). While the patient's gestational diabetes may have indirectly contributed by impairing the inflammatory response and delaying wound repair, its role was likely secondary to the primary surgical and infectious insults (12).

Management of a uterocutaneous fistula depends on the patient's clinical condition and the presence of acute infection. For stable patients without severe infection signs, conservative treatment involving targeted intravenous antibiotics and close observation may be appropriate (5). However, conservative measures alone seldom lead to permanent resolution, as the underlying anatomical defect persists. Surgical removal of the fistulous tract is the definitive treatment, typically performed through an open abdominal procedure (laparotomy) to dissect and excise the entire tract, followed by a double-layer closure to repair the uterine defect (5, 8). Minimally invasive laparoscopic approaches have also shown success in carefully chosen cases (8). Additionally, medical management with gonadotropin-releasing hormone (GnRH) agonists has been investigated as a conservative approach to suppress menstruation and assist in fistula closure for select patients (11, 13). Given that the patient had completed her family and presented with a complex, persistent fistula, a total hysterectomy was selected as the definitive surgical management to ensure complete excision and prevent recurrence (9, 16). This approach was justified by the failure of conservative measures and the need for a permanent resolution of the patient's chronic symptoms (10). Long-term follow-up demonstrated a successful outcome with no recurrence, reinforcing the effectiveness of definitive surgery in cases where conservative management is inappropriate or unsuccessful (2).

To prevent recurrence, it's essential to ensure complete excision of the fistulous tract and careful repair of the uterus. Generally, short-term outcomes after surgical intervention are favorable, with many patients experiencing symptom relief and healing of the fistula (5). Postoperative MRI, typically conducted 1–2 months after surgery, usually confirms closure of the fistula and restoration of normal anatomy. Long-term follow-up data indicate that recurrence is rare when complete excision is achieved (5).

For women in their reproductive years, it is important to consider how both the utero-cutaneous fistula and its surgical repair might impact future fertility and pregnancy outcomes. A uterine scar could potentially raise the risk of complications in subsequent pregnancies, such as abnormal placental development or uterine rupture, although specific data on these risks are limited due to the rarity of the condition (14).

Preventing uterocutaneous fistula begins with precise surgical techniques during cesarean deliveries. This includes careful bleeding control, thorough closure of the hysterotomy using appropriate absorbable sutures, and avoiding unnecessary manipulation of the uterus (5). Prophylactic antibiotics should be administered according to existing guidelines to reduce the chance of postoperative infection (15). Clinicians need to maintain a high level of suspicion for utero-cutaneous fistula in patients who experience ongoing wound discharge following cesarean delivery. Early detection, appropriate imaging—preferably MRI—and a multidisciplinary approach are critical to minimizing patient suffering and achieving effective management of this rare but serious complication (5, 9).

## Conclusion

Uterocutaneous fistula represents a rare yet notable complication that can arise following cesarean delivery, particularly among individuals with a history of multiple cesarean sections and conditions related to placenta accreta. The presence of persistent discharge from a cesarean scar that remains unresponsive to antibiotic treatment should prompt clinical consideration of this condition. Timely identification and the use of appropriate imaging techniques—most notably magnetic resonance imaging—are vital for achieving an accurate diagnosis. Definitive management typically necessitates surgical intervention, and a collaborative approach is essential to ensure the best possible patient outcomes and to mitigate the risk of additional complications.

Prompt intervention can markedly diminish the likelihood of severe morbidity linked to these conditions. Furthermore, educating patients regarding the signs and symptoms to observe postoperatively is essential for early identification and management.

## Informed consent

Written informed consent was obtained from a legally authorized representative(s) for anonymized patient information and images to be published in this article.

## Data Availability

The datasets presented in this article are not readily available because of ethical and privacy restrictions. Requests to access the datasets should be directed to the corresponding authors.

## References

[1] World Health Organization. Obstetric fistula: guiding principles for clinical management and programme development. Available online at: https://www.afro.who.int/sites/default/files/2017-06/mps%20Fistula2.pdf (Accessed February 12, 2026) 10.1016/j.ijgo.2007.06.032 17880979

[2] Ramanidevi T Thendral N Harshini J. Scary scar—uterocutaneous fistula following cesarean delivery: a case report. J South Asian Fed Obstet Gynaecol. (2025) 17(3):346–8. 10.5005/jp-journals-10006-2655

[3] Polan ML Sleemi A Bedane MM Lozo S Morgan MA. Obstetric fistula. In: Debas HT Donkor P Gawande A Jamison DT Kruk ME Mock CN, editors. Essential Surgery: Disease Control Priorities (Volume 1), 3rd Edn. Washington (DC): The International Bank for Reconstruction and Development/The World Bank (2015). Available online at: https://www.ncbi.nlm.nih.gov/books/NBK333495/ (Accessed April 2, 2015). 26740991

[4] Thubert T Denoiseux C Faivre E Naveau A Trichot C Deffieux X. Combined conservative surgical and medical treatment of a uterocutaneous fistula. J Minim Invasive Gynecol. (2012) 19(2):244–7. 10.1016/j.jmig.2011.10.010 22381970

[5] Etrusco A Fabio M Cucinella G de Tommasi O Guastella E Buzzaccarini G. Utero-cutaneous fistula after caesarean section delivery: diagnosis and management of a rare complication. Przegl Menopauzalny. (2022) 21(3):214–7. 10.5114/pm.2022.119263 PMC9551359 36254128

[6] Lim PS Shafiee MN Ahmad S Hashim Omar M. Utero-cutaneous fistula after caesarean section secondary to red degeneration of intramural fibroid. Sex Reprod Healthc. (2012) 3(2):95–6. 10.1016/j.srhc.2012.03.002 22578758

[7] Piñas Carrillo A Chandraharan E. Placenta accreta spectrum: risk factors, diagnosis and management with special reference to the Triple P procedure. Womens Health (Lond). (2019) 15:1745506519878081. 10.1177/1745506519878081 31578123 PMC6777059

[8] Shah N Changede P More V. Laparoscopic management of post-cesarean section uterocutaneous fistula. J Obstet Gynaecol India. (2019) 69(4):380–2. 10.1007/s13224-018-1197-2 31391749 PMC6661046

[9] Cicinelli R Cicinelli E Crupano F Vinciguerra M Lamanna B Vimercati A. A rare but troublesome complication of cesarean section: the uterocutaneous fistula. Report of two cases and review of literature. Case Rep Perinat Med. (2022) 11(1):20210057. 10.1515/crpm-2021-0057 40041211 PMC11800659

[10] Jadib A Tabakh H Chahidi El Ouazzani L Kardi O Siwane A Touil N. Utero-cutaneous fistula following cesarean section: a case report. Radiol Case Rep. (2021) 17(1):77–9. 10.1016/j.radcr.2021.09.069 34765065 PMC8571535

[11] Seyhan A Ata B Sidal B Urman B. Medical treatment of uterocutaneous fistula with gonadotropin-releasing hormone agonist administration. Obstet Gynecol. (2008) 111(2 Pt 2):526–8. 10.1097/01.AOG.0000281670.94265.5c 18239009

[12] Brem H Tomic-Canic M. Cellular and molecular basis of wound healing in diabetes. J Clin Invest. (2007) 117(5):1219–22. 10.1172/JCI32169 17476353 PMC1857239

[13] Yadav P Gupta S Singh P Tripathi S. Successful medical management of uterocutaneous fistula. Int J Gynaecol Obstet. (2014) 124(3):263–4. 10.1016/j.ijgo.2013.09.024 24405990

[14] Yesiladali M Saridogan E Saridogan E. Successful pregnancy and delivery following surgical treatment of postmyomectomy uterocutaneous fistula. BMJ Case Rep. (2019) 12(12):e231594. 10.1136/bcr-2019-231594 31811107 PMC6904194

[15] Laganà AS Cromi A Tozzi R Franchi M Lukanović D Ghezzi F. Uterine scar healing after cesarean section: managing an old surgery in an evidence-based environment. J Investig Surg. (2019) 32(8):770–2. 10.1080/08941939.2018.1465145 29741973

[16] National Cancer Institute. Fistula (2011). Available online at: https://www.cancer.gov/publications/dictionaries/cancer-terms/def/fistula (Accessed February 12, 2026)

