# Supplementary figures and images for: Uterocutaneous fistula following cesarean section: a case report
Source: Front Reprod Health. 2026 Jun 4;8:1847520. doi: 10.3389/frph.2026.1847520 (PMC13275667; doi:10.3389/frph.2026.1847520)

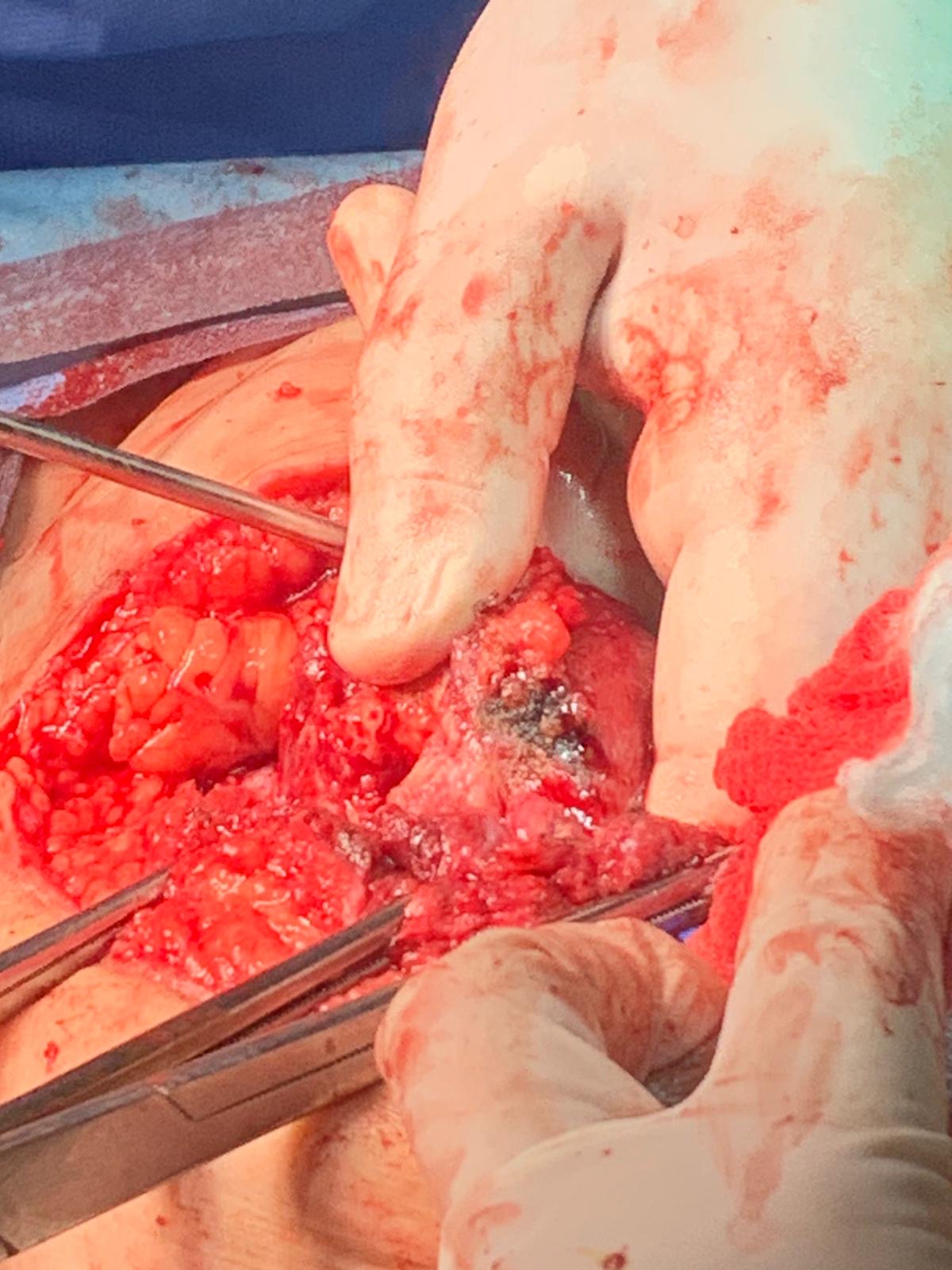

Supplement: Supplementary file 1 [file Image1.jpeg]

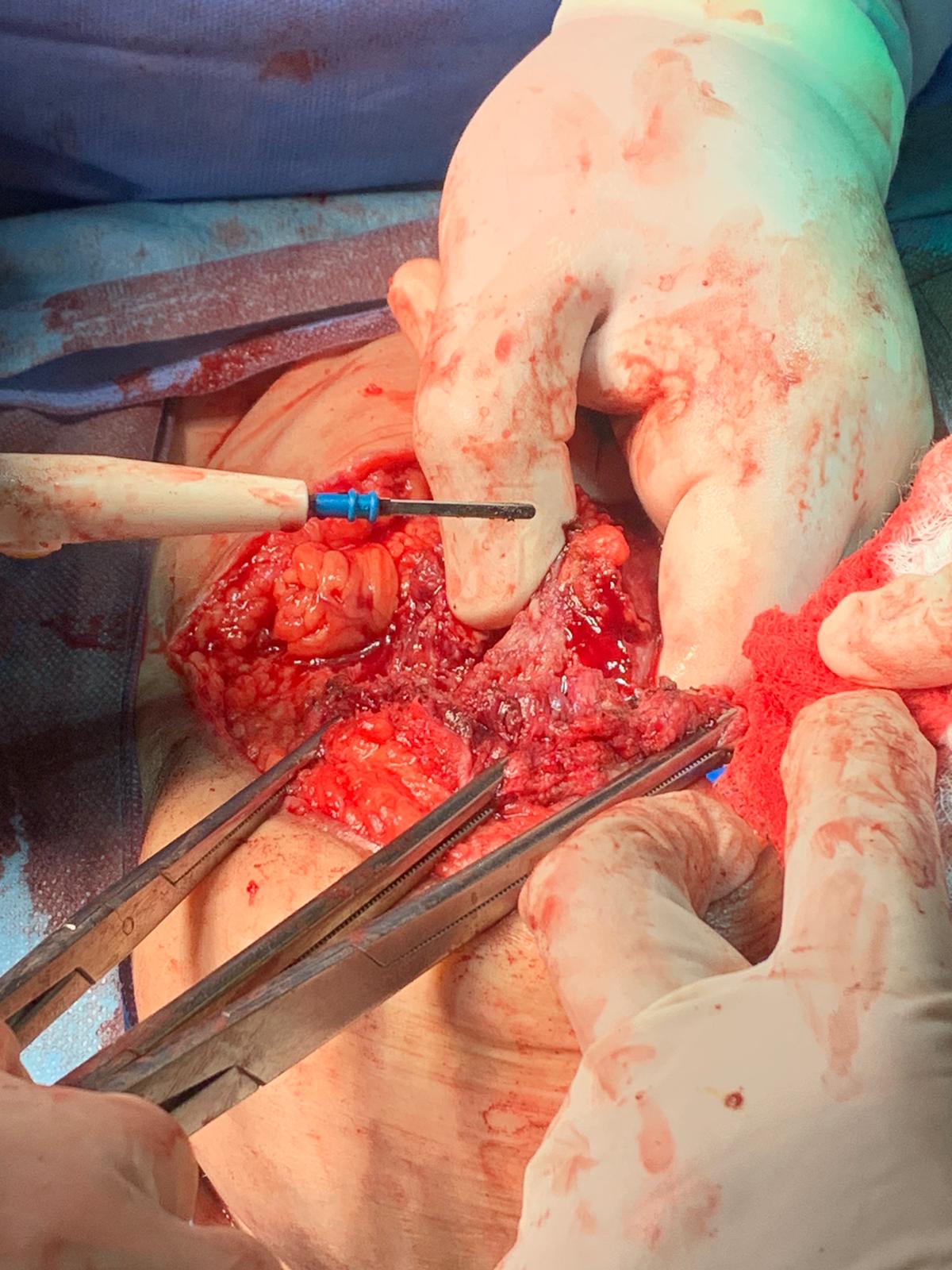

Supplement: Supplementary file 2 [file Image2.jpeg]

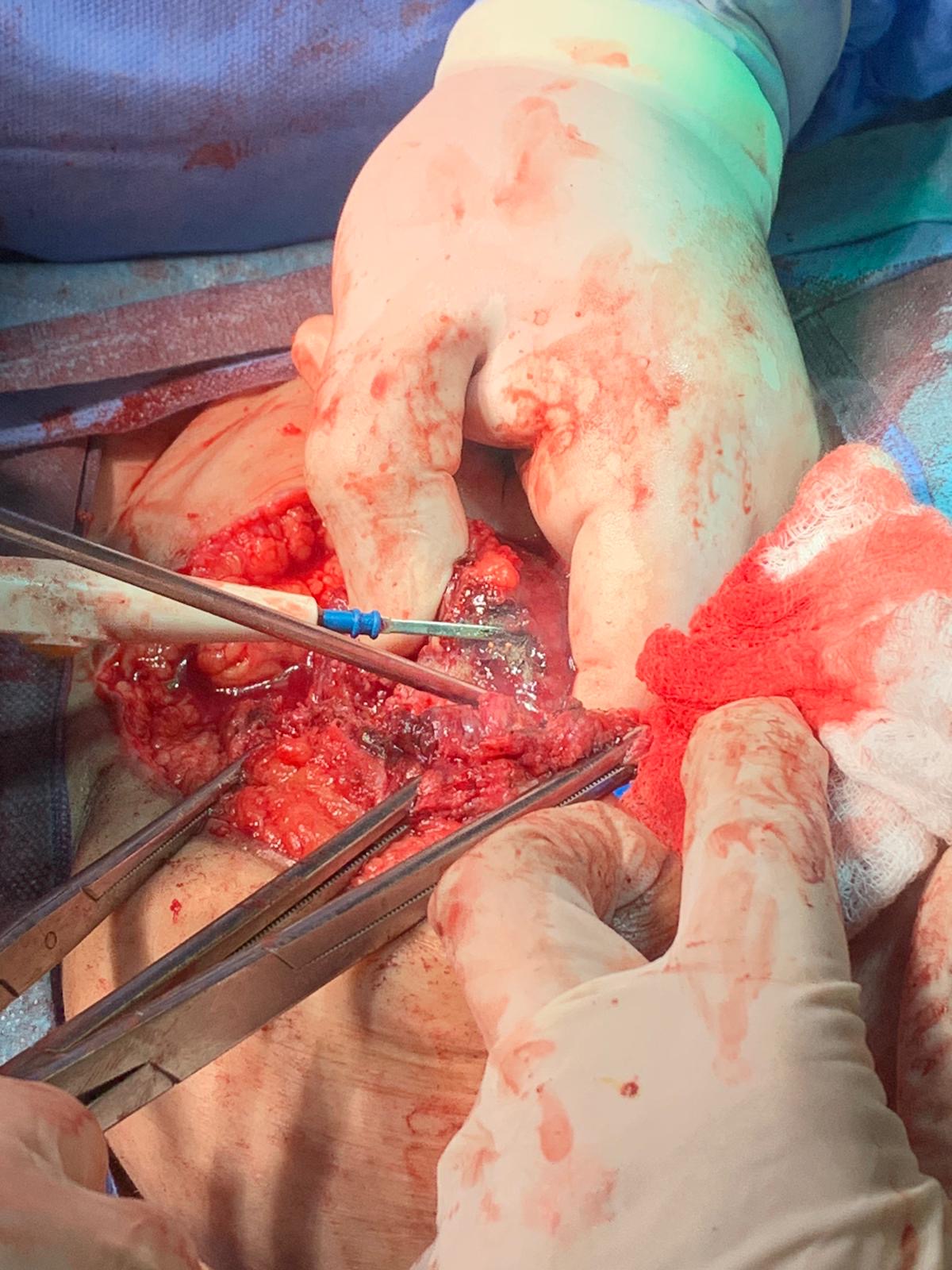

Supplement: Supplementary file 3 [file Image3.jpeg]
